# Avacopan-Assisted Remission of Diffuse Alveolar Hemorrhage in Myeloperoxidase-Antineutrophil Cytoplasmic Antibody-Associated Vasculitis: A Case Report

**DOI:** 10.7759/cureus.107276

**Published:** 2026-04-18

**Authors:** Mahmood Yassin Alattas, Abdullah Mohammad Albihani, Asma Mohammed Almutairi, Ghalia Abdullah Alarwi, Rawan Naif Alraddadi

**Affiliations:** 1 Department of Rheumatology, King Salman Bin Abdulaziz Medical City, Medina, SAU; 2 Department of Internal Medicine, King Salman Bin Abdulaziz Medical City, Medina, SAU

**Keywords:** anca-associated vasculitis, antineutrophil cytoplasmic antibody (anca), avacopan, diffuse alveolar hemorrhage, steroid dependence

## Abstract

Myeloperoxidase (MPO)-specific antineutrophil cytoplasmic antibody (ANCA)-associated vasculitis (AAV) can present with diffuse alveolar hemorrhage (DAH), a life-threatening pulmonary complication, even in the absence of renal involvement. Relapse and steroid-related toxicity remain significant management challenges in such cases. Avacopan, a selective complement C5a receptor antagonist, has been introduced as a steroid-sparing agent; however, its role in pulmonary-dominant MPO-AAV remains underreported. Here, we describe a 24-year-old woman from Medina, Saudi Arabia, who presented with life-threatening DAH and acute hypoxemic respiratory failure secondary to MPO-AAV. She initially responded to pulse intravenous methylprednisolone and intravenous immunoglobulin but suffered an early relapse during treatment, and rituximab was therefore initiated. Due to steroid dependence and recurrent respiratory decline, avacopan 30 mg twice daily was started as an adjunctive therapy. Following sustained clinical remission for more than six months, corticosteroids were withdrawn, and the patient had no recurrence of DAH or further respiratory compromise. This case highlights the therapeutic value of avacopan as a steroid-sparing adjunct in MPO-AAV with severe pulmonary involvement. This is the first reported case from Saudi Arabia of avacopan use in MPO-AAV with predominantly pulmonary involvement, underscoring the need for regional real-world evidence to guide its optimal use in this challenging phenotype.

## Introduction

Antineutrophil cytoplasmic antibody (ANCA)-associated vasculitides (AAV) comprise small-vessel necrotizing vasculitides, including microscopic polyangiitis (MPA), granulomatosis with polyangiitis (GPA), and eosinophilic granulomatosis with polyangiitis [1]. Perinuclear ANCA (P-ANCA) directed against myeloperoxidase (MPO) is commonly linked with MPA and may present with pulmonary involvement, such as diffuse alveolar hemorrhage (DAH), with or without renal disease. DAH affects up to 10% of patients with AAV and is associated with significant mortality, mainly in those requiring mechanical ventilation [2]. For AAV management, the 2021 American College of Rheumatology (ACR)/Vasculitis Foundation and 2024 Kidney Disease: Improving Global Outcomes (KDIGO) guidelines recommend a standard induction regimen of high-dose glucocorticoids plus rituximab or cyclophosphamide [3,4]. Nevertheless, relapses and glucocorticoid-related side effects, including infection, osteoporosis, and metabolic disturbances, remain major concerns [5].

Avacopan, a selective oral complement C5a receptor antagonist, is a steroid-sparing drug that inhibits complement-mediated neutrophil activation. The pivotal ADVOCATE trial has established its efficacy and superiority over glucocorticoid tapering for sustained remission in AAV; however, patients with pulmonary hemorrhage requiring invasive ventilatory support were explicitly excluded from that trial [6]. Subsequent studies have begun to address this critical gap. For instance, a retrospective multicenter observational study by Chalkia et al. demonstrated resolution of pulmonary hemorrhage in all AAV patients with hypoxic DAH treated with avacopan [5]. A recent case report has further documented its favorable effect even in patients requiring mechanical ventilation or extracorporeal membrane oxygenation (ECMO) support [7]. Nevertheless, real-world evidence in the pulmonary-dominant phenotype remains limited, particularly in the absence of renal involvement. Here, we report a case of steroid-dependent MPO-AAV with DAH in a Saudi woman patient, which was successfully managed with the addition of avacopan.

## Case presentation

**Figure 1:**
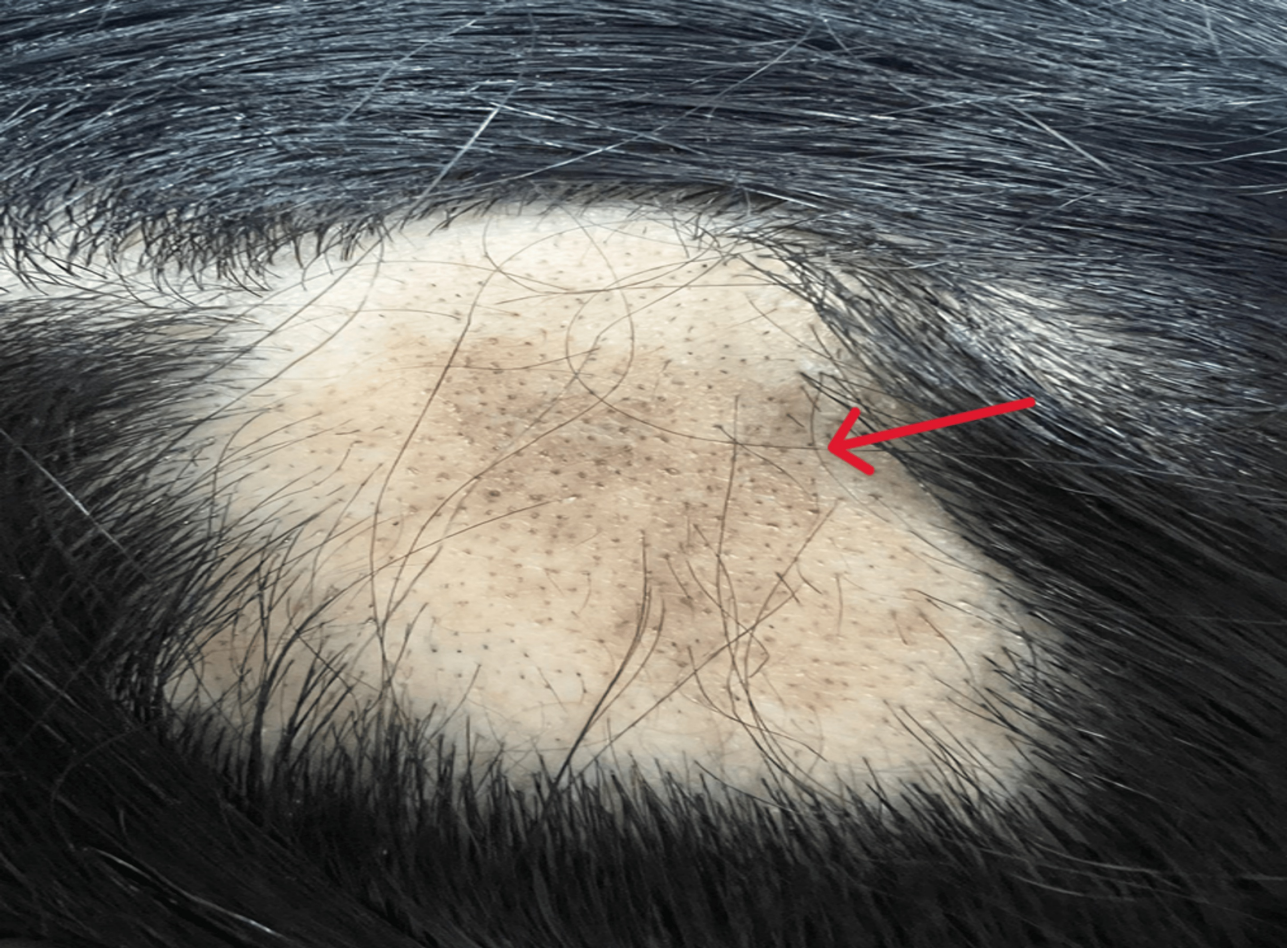
Patient's scalp findings showing patchy alopecia present at diagnosis.

A 24-year-old previously healthy Saudi woman had a four-month history of fatigue, exertional dyspnea, arthralgia, palpitations, patchy non-scarring alopecia (Figure 1), and progressive hearing loss prior to her initial presentation to the Emergency Department (ED). Otolaryngology follow-up showed normal tympanic membranes. However, pure-tone audiometry demonstrated right mild-to-moderate low-frequency conductive hearing loss and left mild-to-moderate mixed hearing loss, suggesting otosclerosis with a sensorineural component. Her past history included iron deficiency anemia, treated with oral supplements. She denied having any rash, photosensitivity, oral or genital ulcers, nasal crusting, or hematuria.

**Figure 2:**
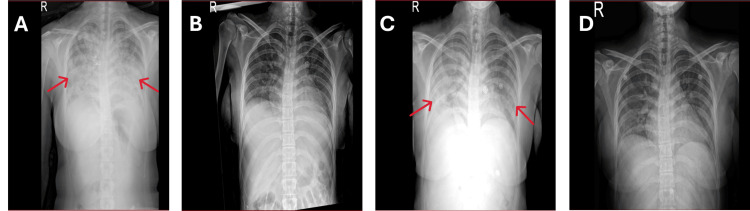
Serial chest radiographs showing the clinical course. (A) Initial presentation with diffuse bilateral airspace opacities. (B) Interval improvement following initial immunosuppressive therapy. (C) Relapse with recurrent bilateral infiltrates. (D) Partial radiographic resolution after treatment with corticosteroids and rituximab.

On December 29, 2024, the patient presented to the Emergency Department with multiple episodes of frank hemoptysis (approximately 100 mL each) and progressive shortness of breath for two days. On arrival, she was in severe respiratory distress with a blood pressure of 138/86 mmHg, heart rate of 121 bpm, respiratory rate of 36 breaths/min, temperature of 37°C, and oxygen saturation of 75% on room air, improving to 95% with a non-rebreathing mask. Chest examination revealed bilateral fine crepitations, and an initial chest X-ray demonstrated diffuse bilateral airspace opacities (Figure 2A).

Laboratory investigations showed leukocytosis at 18.6 × 10⁹/L (normal 4.0-10.0 × 10⁹/L), severe anemia with hemoglobin 6.6 g/dL (normal 12-16 g/dL), platelets 256 × 10⁹/L (normal 150-400 × 10⁹/L), blood urea nitrogen 3.1 mmol/L (normal 2.5-7.5 mmol/L), creatinine 52 µmol/L (normal 45-90 µmol/L), and alanine aminotransferase 7 U/L (normal 7-56 U/L). The direct Coombs test was weakly positive. Autoimmune testing revealed a P-ANCA positive for MPO, a low-titer antinuclear antibody (ANA 1:40), and low complement levels with C3 74.5 mg/dL (normal 79-152 mg/dL) and C4 13.9 mg/dL (normal 16-38 mg/dL) (Table 1). Infectious screening for HIV, hepatitis B and C, acid-fast bacilli, respiratory viruses, and bacterial cultures was negative.

**Table 1:** Summary of laboratory investigations. P-ANCA: perinuclear antineutrophil cytoplasmic antibody.

Test	Result	Reference range
White blood cell count	18.6 × 10⁹/L	4.0-10.0 × 10⁹/L
Hemoglobin	6.6 g/dL	12-16 g/dL
Platelet count	256 × 10⁹/L	150-400 × 10⁹/L
Blood urea nitrogen	3.1 mmol/L	2.5-7.5 mmol/L
Serum creatinine	52 µmol/L	45-90 µmol/L
Alanine aminotransferase	7 U/L	7-56 U/L
Direct Coombs test	Weakly positive	Negative
P-ANCA for myeloperoxidase	Positive	Negative
Antinuclear antibody	1:40 (low titer)	<1:40
Complement C3	74.5 mg/dL	79-152 mg/dL
Complement C4	13.9 mg/dL	16-38 mg/dL

**Figure 3:**
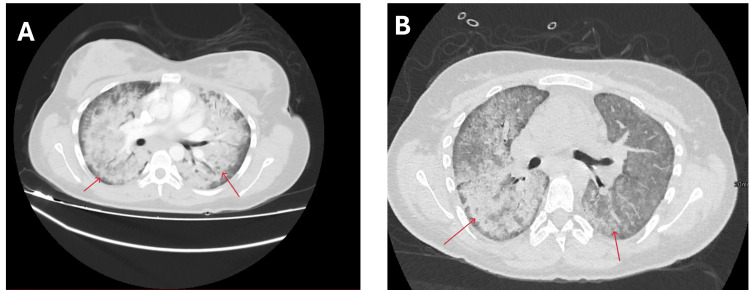
Axial chest CT images. (A) Initial scan at presentation demonstrating diffuse bilateral consolidations and centrilobular nodules, consistent with active diffuse alveolar hemorrhage. (B) Scan during disease relapse, showing recurrence of diffuse alveolar hemorrhage.

CT pulmonary angiography showed diffuse bilateral consolidations with centrilobular nodules consistent with DAH (Figure 3A). Bronchoscopy confirmed DAH with tracheal blood clots and no endobronchial lesion or active bleeding source. Echocardiography revealed mild rheumatic mitral stenosis (mean gradient 5 mmHg) with preserved ventricular function.

Disease activity at presentation was quantified using the Birmingham Vasculitis Activity Score version 3 (BVAS v3) [8], yielding a score of 10, reflecting active arthralgia (one point), sensorineural and conductive hearing loss (three points), and massive alveolar hemorrhage (six points), consistent with severe active disease.

The patient was then admitted to the intensive care unit with acute hypoxemic respiratory failure secondary to DAH, where she was intubated and managed using lung-protective mechanical ventilation. Treatment included pulse methylprednisolone 1 g IV daily for five days, followed by 60 mg IV daily, meropenem, linezolid, blood transfusions, and stress ulcer prophylaxis. Given the critical illness setting with empirical broad-spectrum antimicrobial coverage for suspected superimposed infection and the need to defer rituximab pending hepatitis B serology, intravenous immunoglobulin (IVIG) 0.4 g/kg daily for five days was administered as adjunctive bridging immunomodulation. The patient's condition improved, and she was successfully extubated. A follow-up chest X-ray confirmed radiologic improvement (Figure 2B). She was discharged on January 15, 2025, on a regimen of prednisolone 30 mg orally (PO) daily, bisoprolol 2.5 mg PO daily, and benzathine penicillin 1.2 million units intramuscularly (IM) every four weeks for rheumatic prophylaxis.

At outpatient review on January 27, 2025, she remained stable, awaiting hepatitis B serology before planned rituximab initiation. The following day, she presented with worsening dyspnea and palpitations but without hemoptysis. Oxygen saturation was 78% on room air, improving to 96% with supplemental oxygen. A repeat chest X-ray demonstrated recurrent bilateral infiltrates (Figure 2C), and a CT chest showed recurrent DAH (Figure 3B). Consequently, she received pulse methylprednisolone 1 g IV daily for three days and rituximab 1 g IV. Her condition clinically improved, and intubation was not required. She was discharged on February 2, 2025, on a regimen of amlodipine 5 mg PO daily, bisoprolol 5 mg PO daily, and prednisolone 60 mg PO daily for 14 days. The discharge chest X-ray demonstrated partial radiological resolution (Figure 2D).

**Figure 4:**
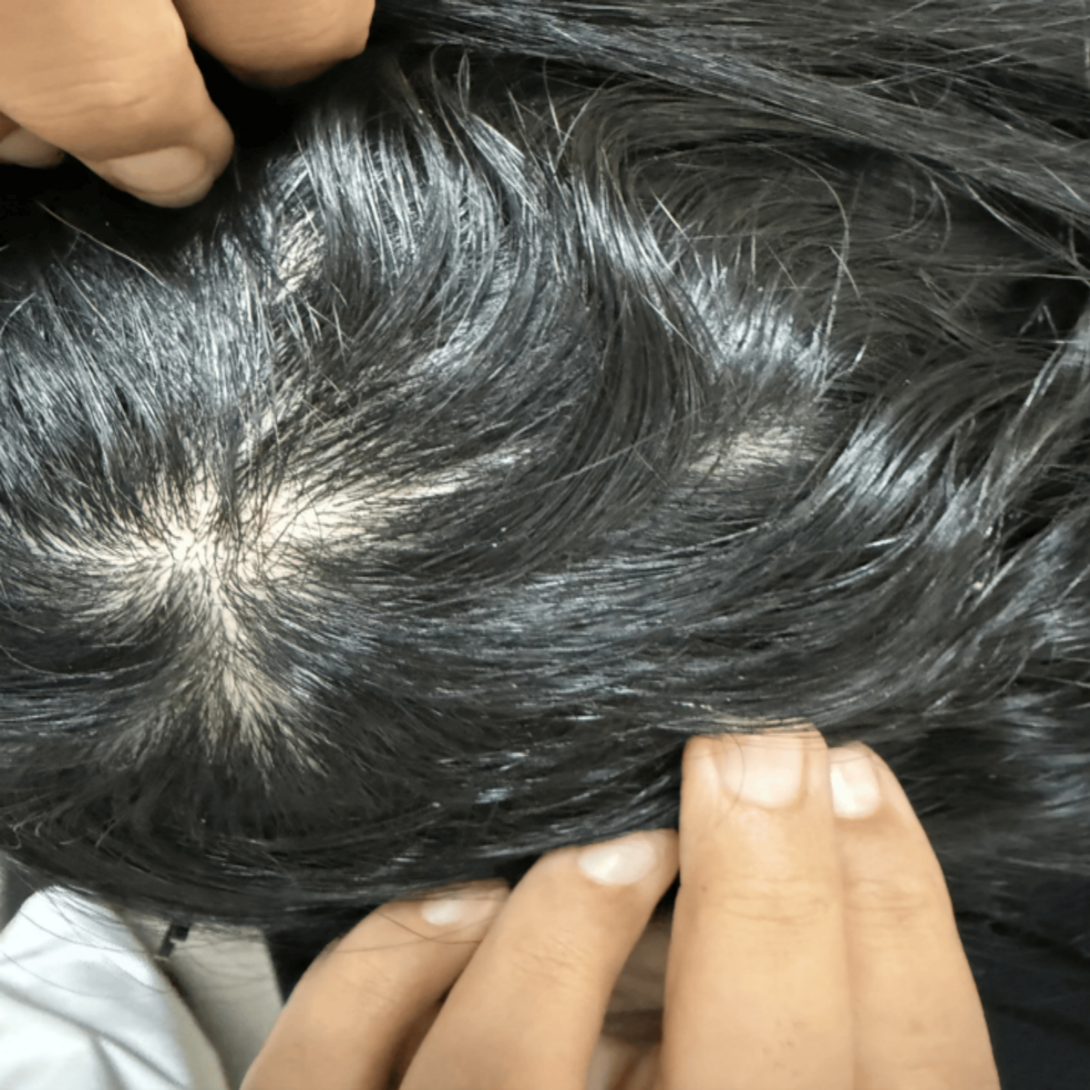
Patient's scalp showing marked regrowth after a six-month course of avacopan therapy.

Due to steroid dependence and pulmonary relapse, avacopan 30 mg PO twice daily was initiated on March 26, 2025. The patient subsequently achieved sustained remission for over six months, and her symptoms, including fatigue and arthralgia, resolved. Also, the alopecia after starting avacopan improved, with significant regrowth observed (Figure 4). Laboratory monitoring showed no elevation in liver enzymes. Importantly, the clinical course was marked by no recurrence of DAH and the successful discontinuation of glucocorticoids.

## Discussion

This report describes a 24-year-old Saudi woman patient with life-threatening pulmonary hemorrhage secondary to MPO-AAV. The diagnosis of AAV was established according to Chapel Hill Consensus Conference definitions [9], while the diagnosis of diffuse alveolar hemorrhage was based on established clinical, radiologic, and bronchoscopic criteria, including the presence of hemoptysis, diffuse bilateral airspace opacities, a falling hemoglobin, and bronchoscopic confirmation of blood-stained airways without an endobronchial source [10]. The case was notable for its demographic atypicality, as AAV typically manifests in the sixth or seventh decade of life [1]. Furthermore, the phenotype was uncommon, characterized by predominant pulmonary involvement without renal dysfunction, which is frequently observed in AAV.

Beyond pulmonary involvement, this case demonstrated two extra-pulmonary manifestations: patchy non-scarring alopecia and progressive asymmetric hearing loss. Alopecia, though not a classic diagnostic criterion for AAV, has been reported as a cutaneous manifestation of systemic autoimmune small-vessel inflammation, likely reflecting microvascular disruption of dermal structures and follicular microvasculature. Crucially, in our patient, alopecia preceded any immunosuppressive therapy, excluding treatment-induced causes such as corticosteroids, and its subsequent resolution following avacopan-mediated remission supports the interpretation that it is a disease manifestation rather than an incidental finding.

Regarding hearing loss, sensorineural and mixed hearing loss are increasingly recognized as audiovestibular manifestations of AAV, particularly in ANCA-positive patients. A previous study by Vainutiene et al. demonstrated audiometric hearing loss in up to 50% of patients with small-vessel vasculitis, with significantly higher prevalence among ANCA-positive individuals [11]. In our patient, the initial otolaryngologic assessment suggested possible otosclerosis; however, this diagnosis is argued by several features, including the asymmetric pattern with differing loss types between ears (conductive right, mixed left), the absence of the characteristic symmetric low-frequency conductive pattern of classic otosclerosis, and the confirmed MPO-ANCA positivity. Rather than reflecting a structural cochlear process, the hearing loss in this case may represent early cochlear microvasculitis, a well-documented but under-recognized manifestation of MPA. Audiologic reassessment following sustained systemic remission would be informative in definitively distinguishing vasculitic from structural etiologies in future follow-up.

Following international management guidelines [3,4], the initial therapeutic regimen, in our case, incorporated glucocorticoids alongside IVIG to suppress inflammation and induce immunomodulation. Partial remission was achieved; however, this conventional regimen failed to sustain durable disease control as the patient experienced a severe relapse within 12 days of hospital discharge. The rapidity of this relapse, despite high-dose maintenance glucocorticoids, is characteristic of steroid-resistant or steroid-dependent disease. A possible mechanistic explanation is that although glucocorticoids, which have broad anti-inflammatory effects, do not specifically inhibit the alternative complement pathway. Consequently, they may be insufficient to suppress complement-mediated neutrophilic activation in patients with intense C5a-driven inflammatory amplification, a pathway recognized as central to AAV pathogenesis [12].

Managing relapses typically requires the intensification of immunosuppression with rituximab dosing and closer monitoring of disease activity [3]. This aligns with our plan, as we started rituximab after the results for hepatitis B were obtained. Although cyclophosphamide represents an equally guideline-endorsed induction alternative, rituximab was preferred given its established superiority in relapsing AAV and the avoidance of cyclophosphamide-associated gonadotoxicity in this 24-year-old patient of reproductive age. However, due to steroid dependence, we introduced avacopan, a selective oral C5a receptor inhibitor. As a steroid-sparing therapy, it directly interrupts the complement amplification loop, a key driver of pathogenesis in AAV. This mechanism is preferred over that of both glucocorticoids and rituximab, as it inhibits neutrophilic inflammation without causing generalized immunosuppression [6].

The clinical course following avacopan initiation was remarkable because the patient achieved sustained clinical remission over the ensuing six months without recurrence of DAH. Crucially, glucocorticoids were successfully discontinued, achieving a state of complete steroid-free remission. This outcome aligns with the evolving therapeutic paradigm promoting complement inhibition and glucocorticoid minimization in high-risk pulmonary MPO-AAV. However, continued longitudinal monitoring with a multidisciplinary approach remains crucial to ensure sustained remission and early detection of recurrence. Moreover, the significant regrowth of alopecia following avacopan initiation served as a clinical marker of sustained systemic remission and improved quality of life, consistent with the interpretation of alopecia as an active disease manifestation discussed above.

The clinical progression of our case is consistent with findings from the ADVOCATE trial, which established the superiority of avacopan over glucocorticoid tapering for sustained remission at week 52 [6]. However, patients requiring mechanical ventilation were excluded from that trial, creating an evidence gap regarding the drug efficacy in patients with severe hypoxic DAH, like ours. A multicenter observational study conducted by Chalkia et al. (2024) [5] addressed this gap. In their cohort, eight patients with AAV requiring respiratory support, pulmonary hemorrhage resolved in all cases following avacopan initiation. Notably, five patients were MPO-ANCA positive, the same serology as the present case. Similarly, avacopan achieved sustained remission in a mechanically ventilated DAH patient requiring ECMO, an even more severe presentation than ours, with successful prednisolone discontinuation within six months, closely mirroring our outcome [7].

The success of C5aR inhibition in this patient has practical implications. Rather than viewing avacopan solely as an alternative to glucocorticoids, it may be more accurate to conceptualize it as complementary to rituximab. By targeting both arms of the immune response simultaneously, adaptive (via rituximab) and innate complement-driven (via avacopan), superior disease control may be achieved compared to rituximab plus glucocorticoids. Nevertheless, more studies are required to validate this hypothesis.

Internationally, a small but growing number of studies have described the use of avacopan in AAV, predominantly from European and North American centers [13]. In Saudi Arabia, previous studies have been conducted to evaluate AAV patterns and outcomes [14], but no local case reports have described the use of avacopan for AAV or its associated DAH. Therefore, to our knowledge, this is the first reported Saudi case demonstrating successful incorporation of avacopan into the treatment of relapsing pulmonary MPO-AAV. However, this may not generalize to all patients with AAV. Geographic, genetic, or demographic factors may influence disease severity or treatment responsiveness. Thus, prospective, controlled studies in diverse populations are necessary to define the precise role of avacopan in different AAV phenotypes.

## Conclusions

Pulmonary-dominant MPO-AAV can relapse despite appropriate immunosuppressive therapy, and managing recurrent DAH remains particularly challenging. This case illustrates that complement pathway inhibition with avacopan may represent a valuable adjunctive therapy in steroid-dependent AAV with severe pulmonary involvement, enabling sustained remission and glucocorticoid withdrawal in a patient who had failed conventional therapy. Although findings from a single case cannot be generalized, this report contributes to the emerging evidence supporting avacopan's role in pulmonary-dominant AAV and underscores the need for prospective studies to define its optimal positioning within the treatment plan.
